# Clinical and imaging differentiation of intrapancreatic accessory spleen from pancreatic neuroendocrine tumor and solid-pseudopapillary tumor: a retrospective analysis

**DOI:** 10.3389/fsurg.2026.1943757

**Published:** 2026-09-23

**Authors:** Shiwei Yang, Haonan Liu, Jiaoxing Wu, Fangzhou Wang, Lidan Chang, Cancan Zhou, Zheng Wu, Shuai Wu

**Affiliations:** Department of Hepatobiliary Surgery, The First Affiliated Hospital of Xi'an Jiaotong University, Xi'an, Shaanxi, China

**Keywords:** differential diagnosis, intrapancreatic accessory spleen, pancreatic neuroendocrine tumor, retrospective analysis, solid pseudopapillary tumor

## Abstract

**Background:**

Intrapancreatic accessory spleen (IPAS) represents a rare anatomical variant of the spleen that is misdiagnosed as pancreatic neoplastic lesions. Notably, IPAS is often erroneously identified as pancreatic neuroendocrine tumor (PNET) or solid pseudopapillary tumor (SPT), which consequently results in unnecessary resection of the pancreas. This study aimed to summarize the clinical, imaging and laboratory characteristics of these lesions, clarify the key differential diagnostic points among them, and thereby enhance the clinical understanding and preoperative diagnostic accuracy of IPAS.

**Methods:**

This retrospective study analyzed all 7 cases of IPAS diagnosed at our hospital from January 2008 to December 2025. For comparative analysis, consecutive patients with pathologically confirmed PNET (*n* = 18) and SPT (*n* = 21) treated at the same institution between January 2023 and June 2025 were included. Clinical, imaging and pathologic characteristics were compared descriptively. Key points of differential diagnosis were summarized.

**Results:**

IPAS was distinguished from PNET and SPT primarily by consistent location in the pancreatic tail (100%), frequent heterogeneous arterial-phase enhancement with CT attenuation often similar to the spleen, universal adjacency to the splenic vessels, solid consistency, and predominantly reddish-brown gross appearance, with most lesions showing a soft texture. In contrast, PNET and SPT showed more variable location, enhancement patterns, attenuation relative to spleen, vessel contact rates, and pathologic color/texture. SPTs are typically larger and more frequently exhibit calcification.

**Conclusion:**

For lesions located in the pancreatic tail, adjacent to the spleen, and exhibiting heterogeneous enhancement in the arterial phase, the possibility of IPAS should be thoroughly considered. Nevertheless, surgical resection remains rational and appropriate when preoperative evaluation cannot completely exclude the possibility of PNET, SPT, or other pancreatic lesions.

## 1 Introduction

In the general population, the incidence of accessory spleen is roughly 10%, with intrapancreatic accessory spleen (IPAS) accounting for 20% of all accessory spleens. Accordingly, the incidence of IPAS in the general population is approximately 2% (1). Most patients with IPAS rarely exhibit specific clinical symptoms and are usually found to have pancreatic space-occupying lesions incidentally (2). Moreover, due to the similarity in imaging manifestations among IPAS, pancreatic neuroendocrine tumor (PNET) and solid pseudopapillary tumor (SPT), IPAS can be misdiagnosed as PNET or SPT (3, 4). Such misdiagnosis may lead to unnecessary surgical intervention. Accordingly, it is clinically imperative to systematically summarize the clinical features of IPAS and elucidate the key points for its differentiation from other pancreatic diseases, thereby enhancing preoperative diagnostic accuracy and minimizing the risk of misdiagnosis.

In embryology, accessory spleen is a congenital abnormality caused by the failure of splenic fusion during the embryonic development process (5). The fusion of the spleen and the tail of the pancreas can be explained as follows: the splenic primordium and the dorsal pancreatic bud are very close to each other before the rotation of the stomach and its surrounding organs, thereby forming IPAS (6). In imaging examinations, IPAS usually shows similar characteristics to splenic tissue. On contrast-enhanced computed tomography (CE-CT), both IPAS and normal splenic tissue exhibit almost the same enhancement pattern: heterogeneous enhancement in the early arterial phase (7). However, due to its tissue characteristics of being filled with red blood cells, IPAS is mostly similar to pancreatic vascular-rich tumors such as PNETs or SPTs on CT examination, which increases the risk of misdiagnosis of IPAS (8). In clinical diagnosis and treatment, even if IPAS is considered preoperatively in patients with pancreatic space-occupying lesions, surgical resection remains the preferred treatment when specific diagnostic methods (such as Tc-99m HDRBC scintigraphy or EUS-FNA) are lacking and neoplastic lesions such as PNETs and SPTs cannot be ruled out clearly. A definite diagnosis is made after obtaining pathological results. Notably, since pancreatic surgery may lead to a decrease in quality of life, adequate preoperative patient counseling is essential to avoid patient dissatisfaction or medicolegal disputes (9).

In this study, we systematically reviewed and summarized the IPAS cases in our center, presenting the diagnosis and treatment process as well as the characteristics of clinicopathology and laboratory examinations. We compared and analyzed the imaging characteristics of IPAS with those of PNET and SPT recently treated in our center. This study aims to enhance preoperative diagnosis of IPAS, improve clinical recognition, and avoid medical disputes.

## 2 Patients and methods

### 2.1 Study design and ethics

This retrospective study was conducted at the Department of Hepatobiliary Surgery, First Affiliated Hospital of Xi'an Jiaotong University. The study protocol was approved by the Institutional Ethics Committee (approval number: [No. XJTU1AF2015LSL-057]), and the requirement for informed consent was waived due to the retrospective nature of the study and the use of fully de-identified data, in accordance with the Declaration of Helsinki and local regulatory requirements.

### 2.2 Study population

We retrospectively reviewed and collected clinical data from 7 patients with confirmed IPAS managed at our center between January 2008 and December 2025. Meanwhile, we consecutively enrolled 18 patients with pathologically confirmed PNET and 21 patients with SPT treated at our institution between January 2023 and June 2025. To simulate the actual preoperative diagnostic dilemma posed by IPAS, PNET and SPT inclusion was deliberately restricted to solid lesions ≤ 5 cm in maximal diameter located in the pancreatic body or tail, reflecting the morphologic spectrum that overlaps with IPAS on cross-sectional imaging.

### 2.3 Data collection

For all patients, demographic characteristics, perioperative variables, cross-sectional imaging features, and pathological findings were retrospectively extracted from the electronic medical record system.

### 2.4 Statistical analysis

Given the small sample size and the exploratory, descriptive nature of this study, continuous variables are presented as medians with interquartile ranges (IQRs), and categorical variables as frequencies with percentages. No formal hypothesis testing was performed. Data were analyzed using [SPSS version 27.0, IBM Corp., Armonk, NY, USA].

## 3 Results

### 3.1 Patients' cohort

All 7 patients with confirmed IPAS managed at our center between January 2008 and December 2025 were identified. For comparative analysis, 18 consecutive patients with pathologically confirmed PNET and 21 with SPT, treated at our institution between January 2023 and June 2025, were enrolled. To simulate the actual preoperative diagnostic dilemma posed by IPAS, control inclusion was deliberately restricted to solid lesions ≤5 cm in maximal diameter located in the pancreatic body or tail, reflecting the morphologic spectrum that overlaps with IPAS on cross-sectional imaging. The patient selection and enrollment flowchart is shown in Figure 1.

**Figure 1:**
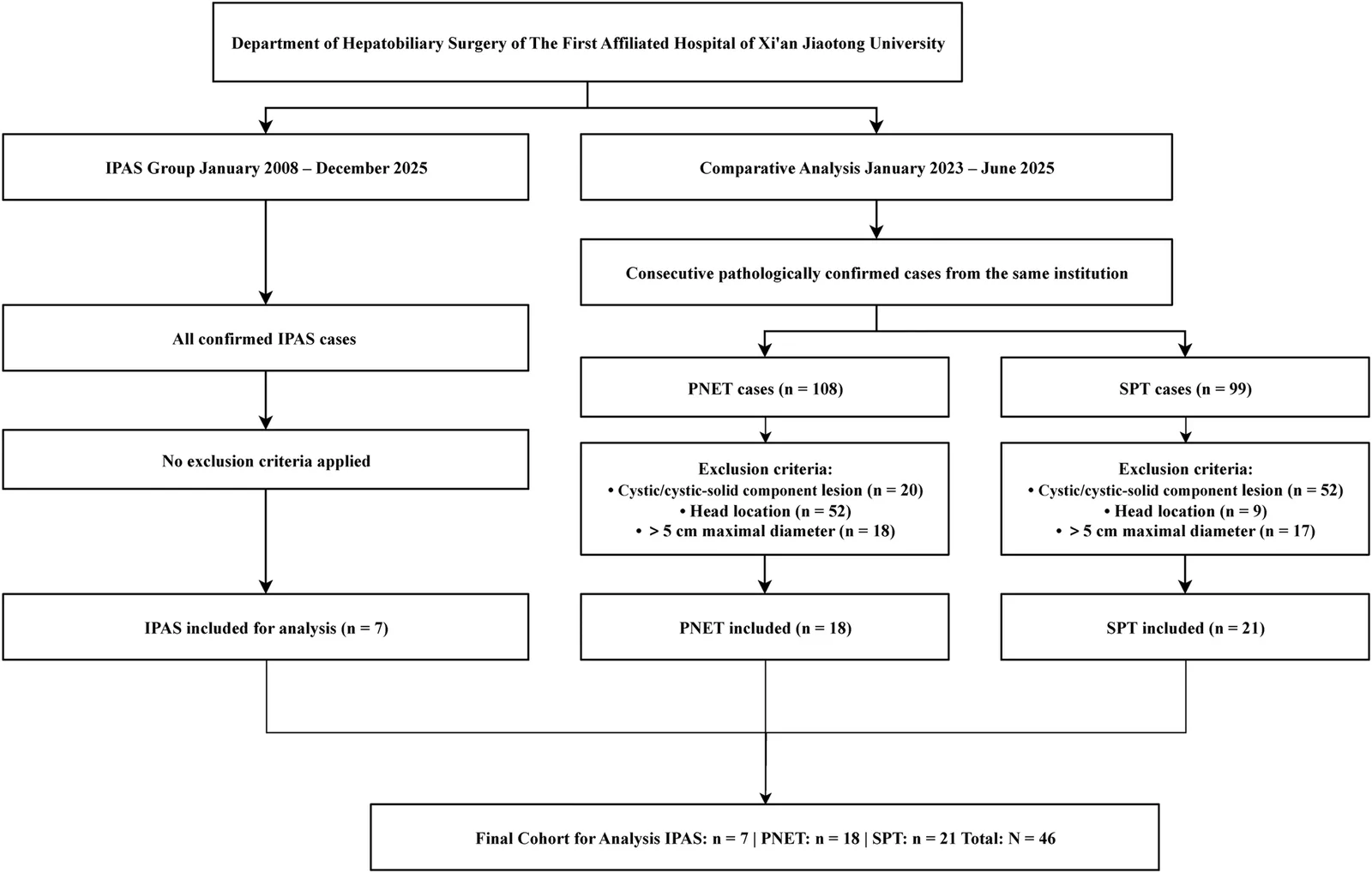
Flow diagram of patient enrollment.

### 3.2 Demographic characteristics of IPAS

Table 1 depicts the baseline demographic characteristics of the IPAS group. The IPAS group enrolled in this study consisted of 4 male and 3 female patients, with ages ranging from 19 to 74 years. The chief complaints of these 7 patients were categorized into four types as follows: two patients were asymptomatic; one presented with dyspnea complicated by jaundice; one suffered from abdominal pain; and three exhibited symptoms highly suspicious of PNET, including hunger-related syncope, lower extremity weakness, and post-exercise dizziness. Except for one patient diagnosed with IPAS who did not undergo surgical intervention, the other six patients all received surgical treatment and were suspected of neuroendocrine tumor before surgery. All six patients who underwent surgical intervention received distal pancreatectomy. Notably, one patient who underwent laparoscopic distal pancreatectomy with splenectomy and cholecystectomy (LDP + S + LC) had an operation duration of 545 min and intraoperative blood loss of 400 mL; in contrast, the remaining patients had both operation duration and intraoperative blood loss below 260 min and 100 mL or less, respectively. During the hospital stay, all patients maintained normal blood glucose levels, which ranged from 4.43 to 5.8 mmol/L. Pathological examination confirmed multiple accessory spleens (≥2) in two patients. Additionally, two other patients were diagnosed with secondary thrombocytosis.

**Table 1:** Perioperative characteristics of patients with IPAS.

ID	Gender	BMI	Age(years)	Main complaint(s)	Comorbid condition(s)	Preoperative diagnosis	Surgical procedure	Operation duration(min)
1	Male	18.7	23	Hunger-induced dizziness, cold sweats, occasional syncope	Secondary adrenocortical insufficiency and subclinical hypothyroidism.	PNET	DP	255
2	Male	23.3	74	Weakness in both lower extremities	Lacunar infarction; type 2 diabetes mellitus.	IPAS	No surgical intervention	NA
3	Male	32.7	73	Scleral icterus, Chest tightness and dyspnea	Stage 2 hypertension	PNET	LDP + S + LC	545
4	Female	18.4	55	Abdominal pain	Hepatic cyst	PNET	LDP + S	130
5	Male	23.3	28	Asymptomatic	Hypertension; chronic nephritis; gout, non-cirrhotic portal hypertension	PNET	RALDP + S + LC	210
6	Female	21.4	19	Post-exercise dizziness and palpitations	None	PNET(Insulinoma)	LSPDP	257
7	Female	23.1	42	Asymptomatic	Hypothyroidism	PNET	LPDP	90

ID	Intraoperative blood loss(mL)	Admission random blood glucose (mmol/L)	Postoperative diagnosis
1	20	5.8	IPAS
2	NA	5.55	IPAS
3	400	5.08	IPAS; Secondary thrombocytosis
4	100	4.82	IPAS; AS adjacent to the spleen
5	100	4.43	IPAS; Secondary thrombocytosis
6	20	4.83	IPAS; AS adjacent to the spleen
7	30	4.84	IPAS

AS, Accessory spleen; BMI, body mass index; DP , Distal pancreatectomy; IPAS, Intrapancreatic Accessory Spleen; LDP + S , Laparoscopic distal pancreatectomy with splenectomy; LDP + S + LC , Laparoscopic distal pancreatectomy with splenectomy and cholecystectomy; LPDP, Laparoscopic partial distal pancreatectomy; LSPDP, Laparoscopic spleen-preserving distal pancreatectomy; PNET, Pancreatic Neuroendocrine Tumor; RALDP + S + LC , Robot-assisted laparoscopic distal pancreatectomy with splenectomy and cholecystectomy.

### 3.3 Imaging features of IPAS

Table 2 summarizes the basic imaging features of the IPAS patients. Among the five patients who received preoperative magnetic resonance imaging (MRI), the signal intensity of IPAS was higher than that of the pancreas on T1-weighted imaging (T1WI) in three cases, while the IPAS signal intensity was comparable to that of the pancreas in the remaining two. In contrast, the signal intensity of IPAS was consistently higher than that of the pancreas on T2-weighted imaging (T2WI) in all five patients. Six out of the seven patients underwent preoperative contrast-enhanced computed tomography (CE-CT). The imaging revealed heterogeneous enhancement in the lesions of all these patients. In four patients who underwent CT attenuation values of both the lesions and the spleen, the CT values of the lesions were essentially consistent with those of the spleen. Figure 2 shows the typical manifestations of MRI and CE-CT in IPAS.

**Table 2:** Imaging characteristics of patients with IPAS.

ID	T1WI signal relative to pancreas	T2WI signal relative to pancreas	CE-CT findings of the mass in arterial phase	Arterial phase CT value of the mass (HU)	Venous phase CT value of the mass (HU)	Arterial phase CT value of spleen (HU)	Venous phase CT value of spleen (HU)
1	NA	NA	NA	NA	NA	NA	NA
2	Higher	Higher	Heterogeneous enhancement	NA	NA	NA	NA
3	Equal	Higher	Heterogeneous enhancement	89	81	89.4	79
4	Higher	Higher	Heterogeneous enhancement	85	145	78	142
5	Equal	Higher	Heterogeneous enhancement	NA	NA	NA	NA
6	Higher	Higher	Heterogeneous enhancement	134	104	128	109
7	NA	NA	Heterogeneous enhancement	104	99	114	99

HU, Hounsfield Unit; T1WI, T1-weighted imaging; T2WI, T2-weighted imaging.

**Figure 2:**
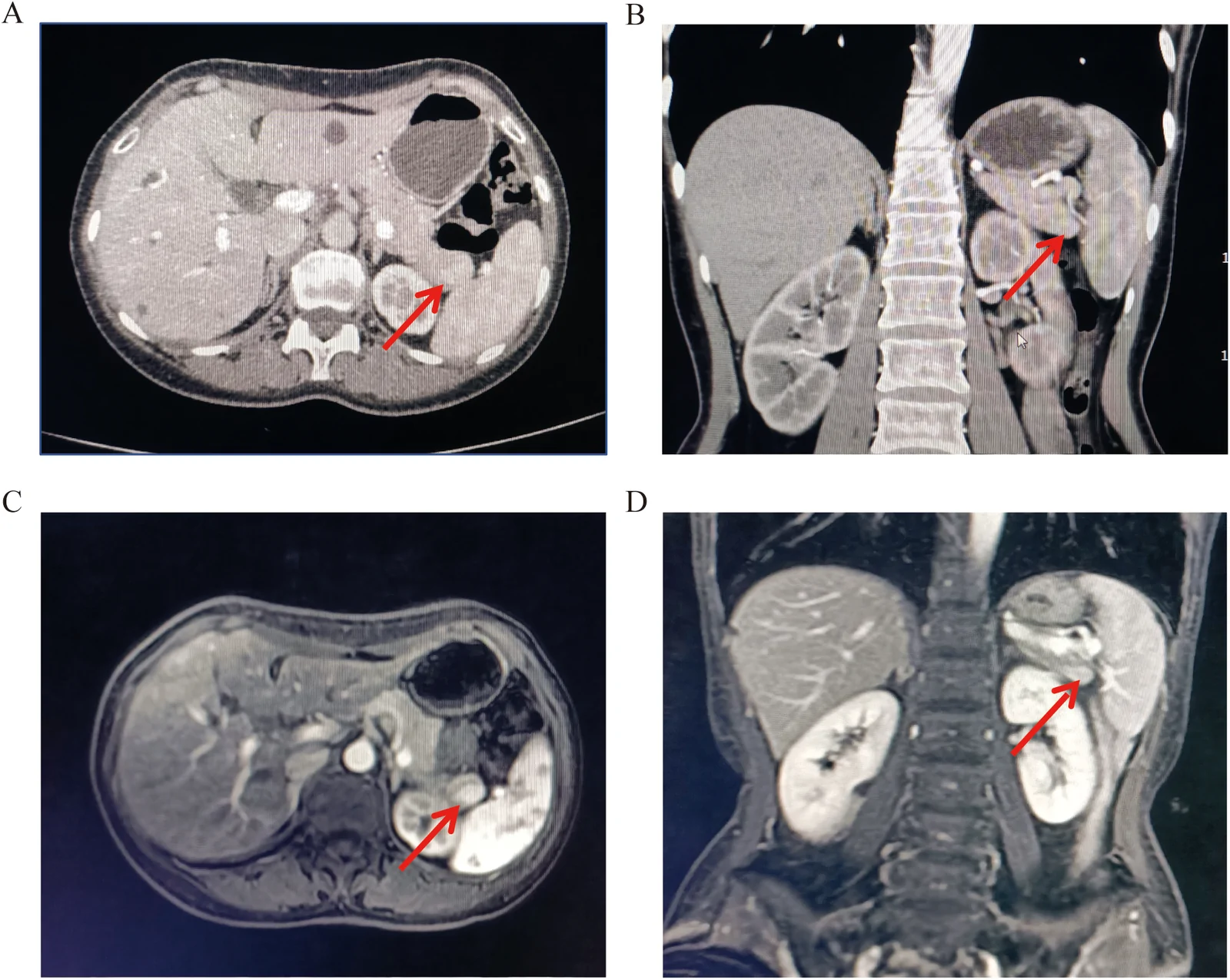
Contrast-enhanced CT and MRI features of IPAS. **(A)** Axial arterial-phase CE-CT shows a well-circumscribed nodule (red arrow) in the pancreatic tail with heterogeneous enhancement matching the spleen. **(B)** Coronal arterial-phase CE-CT clearly demonstrates the intrapancreatic location (red arrow) of the lesion. **(C)** Axial arterial-phase T1-weighted CE-MRI reveals homogeneous hyperenhancement of the lesion (red arrow). **(D)** Coronal arterial-phase T1-weighted CE-MRI confirms the focal lesion (red arrow) within the pancreas. Scale bar = 2 cm.

### 3.4 Morphologic and pathological characteristics of IPAS

Table 3 presents the morphologic characteristics of the seven patients with IPAS. Gross pathological texture was available for the six surgically treated patients: four lesions were soft and two were hard; texture data were unavailable for the non-surgically managed patient. All seven IPAS patients exhibited consistent features: all lesions were confirmed to be solid and well-circumscribed (Figure 3). Regarding the tumor morphology, five of the IPAS lesions appeared as spheroidal, while the remaining two manifested as crescent-shaped and elliptical, respectively. Regarding lesion size, the size of IPAS varied from 13 mm to 50 mm. Regarding the anatomical relationship with adjacent structures, the IPAS lesions in 6 patients were located within 10 mm of the spleen, among which 3 lesions were closely adherent to the splenic parenchyma. All IPAS lesions were invariably situated adjacent to the splenic vessels and did not induce dilation of the pancreatic duct.

**Table 3:** Morphologic characteristics of patients with IPAS.

ID	Texture of the mass	Nature of the mass	Margins of the mass	Shape of the mass	Maximum diameter (mm)	Distance between IPAS and the spleen (mm)	IPAS is adjacent to the splenic vessels	Pancreatic duct dilatation
1	Soft	Solid	Well-circumscribed	Spheroid	30	0	Yes	No
2	NA	Solid	Well-circumscribed	Spheroid	50	3.3	Yes	No
3	Hard	Solid	Well-circumscribed	Spheroid	20	20.5	Yes	No
4	Soft	Solid	Well-circumscribed	Oblate Spheroid	15	0	Yes	No
5	Soft	Solid	Well-circumscribed	Crescent	22.8	4.1	Yes	No
6	Hard	Solid	Well-circumscribed	Spheroid	13	10.0	Yes	No
7	Soft	Solid	Well-circumscribed	Elliptical	13	0	Yes	No

**Figure 3:**
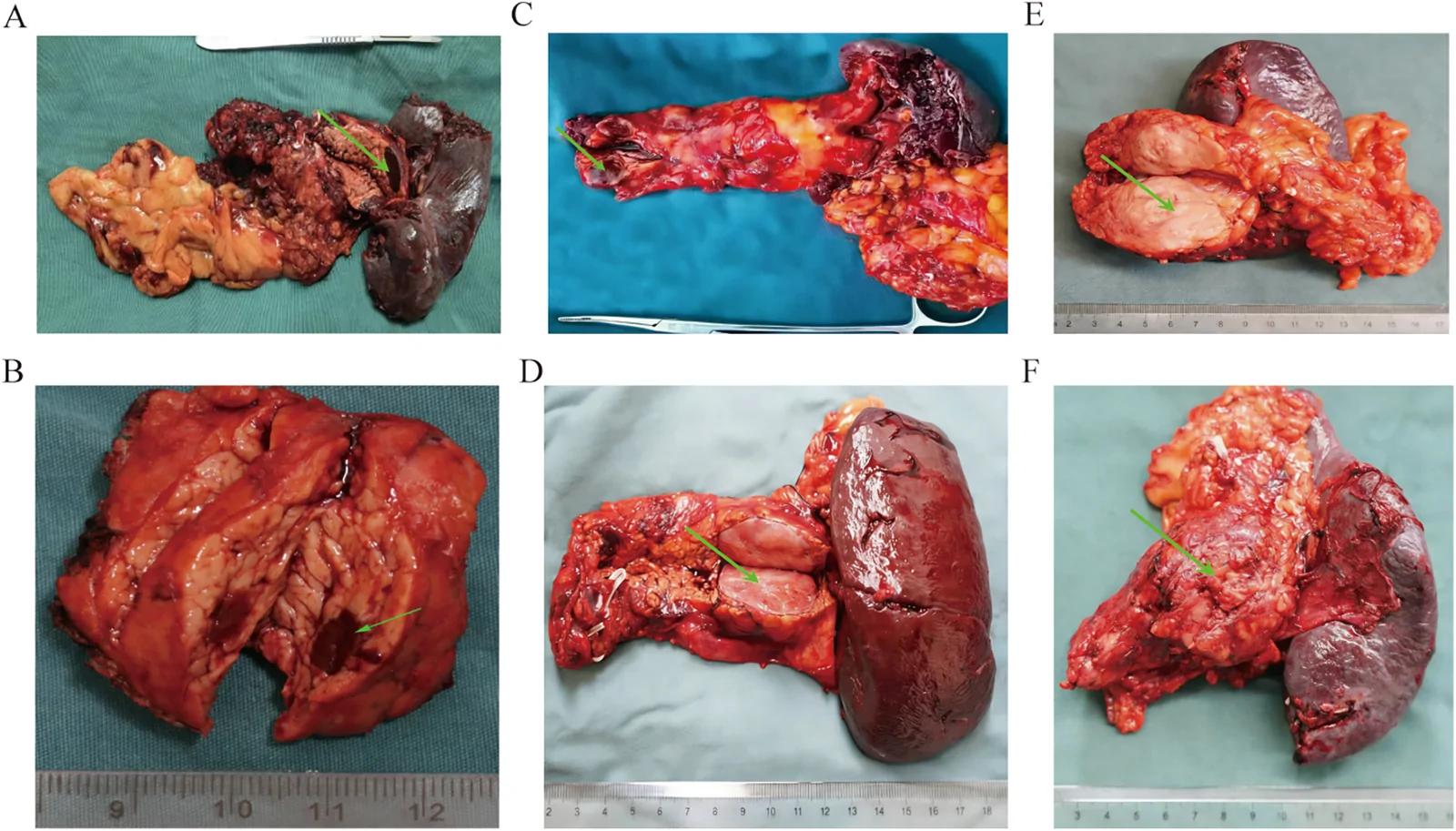
Gross pathological specimens of IPAS, PNET and SPT. Green arrows indicate the lesion sites. Two IPAS cases showing **(A)** reddish-brown and **(B)** reddish-brown appearance. Two PNET cases showing **(C)** reddish-brown and **(D)** gray-white appearance. Two SPT cases showing **(E)** gray-white and **(F)** reddish-brown appearance.

Tables 4, 5 present a descriptive comparison of the imaging and pathological characteristics of IPAS (*n* = 7), PNET (*n* = 18), and SPT (*n* = 21).

**Table 4:** Imaging characteristics of IPAS, SPT, and PNET.

Characteristic	Category	IPAS (*n* = 7)	SPT (*n* = 21)	PNET (*n* = 18)
Lesion location characteristics				
	Tail	7 (100.0%)	14 (66.7%)	13 (72.2%)
	Body	0 (0.0%)	7 (33.3%)	5 (27.8%)
	Calcification			
	Present	0 (0.0%)	12 (57.1%)	0 (0.0%)
	Absent	7 (100.0%)	9 (42.9%)	18 (100.0%)
Imaging lesion size [cm, median (IQR)]		2.0 (1.4–2.6)	3.2 (2.5–3.9)	1.7 (1.4–2.4)
Arterial-phase enhancement pattern				
	Homogeneous	0 (0.0%)	3 (14.3%)	6 (33.3%)
	Heterogeneous	6 (85.7%)	18 (85.7%)	12 (66.7%)
	Not reported	1 (14.3%)	0 (0.0%)	0 (0.0%)
CT attenuation relative to spleen				
	Higher than spleen	1 (14.3%)	0 (0.0%)	13 (72.2%)
	Similar to spleen	2 (28.6%)	0 (0.0%)	0 (0.0%)
	Lower than spleen	1 (14.3%)	21 (100.0%)	5 (27.8%)
	Not reported	3 (42.9%)	0 (0.0%)	0 (0.0%)
Splenic-vessel adjacency/contact				
	Yes/contact or adjacent	7 (100.0%)	10 (47.6%)	11 (61.1%)
	No explicit contact/adjacency	0 (0.0%)	11 (52.4%)	7 (38.9%)
Well-defined margin				
	Yes	7 (100.0%)	20 (95.2%)	13 (72.2%)
	No	0 (0.0%)	1 (4.8%)	5 (27.8%)
Pancreatic duct dilation				
	Present	0 (0.0%)	0 (0.0%)	0 (0.0%)
	Absent	7 (100.0%)	21 (100.0%)	18 (100.0%)

Continuous variables are presented as median (IQR). No hypothesis tests or *P* values were calculated; the table is descriptive only.

**Table 5:** Pathologic characteristics of IPAS, SPT, and PNET.

Characteristic	Category	IPAS (*n* = 6)	SPT (*n* = 21)	PNET (*n* = 18)
Maximum diameter [cm, median (IQR)]		1.75 (1.4–2.1)	3.0 (2.6–4.5)	1.6 (1.4–2.5)
Tumor texture				
	Soft	4 (66.7%)	4 (19.0%)	2 (11.1%)
	Intermediate	0 (0.0%)	7 (33.3%)	9 (50.0%)
	Hard	2 (33.3%)	10 (47.6%)	7 (38.9%)
Tumor color				
	Red/brown	6 (100.0%)	6 (28.6%)	7 (38.9%)
	Gray/white/yellow	0 (0.0%)	9 (42.9%)	8 (44.4%)
	Mixed red/brown + gray/white/yellow	0 (0.0%)	6 (28.6%)	3 (16.7%)
Well-defined margin				
	Yes	6 (100.0%)	20 (95.2%)	13 (72.2%)
	No	0 (0.0%)	1 (4.8%)	5 (27.8%)

Continuous variables are presented as median (IQR). No hypothesis tests or *P* values were calculated; the table is descriptive only.

Pathology denominators for IPAS are 6 for texture, color, margin and pathologic size, because pathological data were not available for all 7 records. No missing pathology data were imputed.

### 3.5 Comparative analysis between IPAS and PNET

Regarding lesion location, all IPAS lesions in this cohort were located in the pancreatic tail (7/7); PNETs were located in the pancreatic tail in 13 cases (72%) and in the pancreatic body in 5 cases (28%). For arterial-phase enhancement pattern on contrast-enhanced CT (evaluable in 6/7 IPAS cases; 1 case was excluded due to poor image quality), IPAS showed heterogeneous enhancement in 6 cases; PNETs showed homogeneous enhancement in 6/18 (33%) and heterogeneous enhancement in 12/18 (67%). For CT attenuation relative to the spleen (evaluable in 4/7 IPAS cases; 3 cases had missing data), IPAS showed attenuation similar to the spleen in 2/4 cases, higher than the spleen in 1/4, and lower than the spleen in 1/4; PNETs showed attenuation higher than the spleen in 13/18 (72%) and lower than the spleen in 5/18 (28%). For splenic-vessel adjacency, all IPAS lesions in this cohort were adjacent to splenic vessels (7/7); PNETs showed splenic-vessel contact in 11/18 (61%). Regarding margin definition, all IPAS lesions in this cohort had well-defined margins (7/7); PNETs had well-defined margins in 13/18 (72%). No pancreatic duct dilation was observed in either group in this cohort. For pathological maximum diameter, the median was 1.75 cm [interquartile range (IQR), 1.4–2.1] for IPAS and 1.6 cm (IQR, 1.4–2.5) for PNETs. Regarding consistency, IPAS lesions were soft in 4/6 cases; PNETs were soft in 2/18 (11%), intermediate in 9/18 (50%), and hard in 7/18 (39%). For gross color, all IPAS lesions in this cohort were reddish-brown (6/6); PNETs were reddish-brown in 7/18 (39%), grayish-white or yellow in 8/18 (44%), and mixed in 3/18 (17%). Regarding pathological margin definition, all IPAS lesions in this cohort had well-defined margins (6/6); PNETs had well-defined margins in 13/18 (72%). For imaging-measured lesion size, the median was 2.0 cm (IQR, 1.4–2.6) for IPAS and 1.7 cm (IQR, 1.4–2.4) for PNETs. No calcification was observed in either group in this cohort.

### 3.6 Comparative analysis between IPAS and SPT

For lesion location, all IPAS lesions in this cohort were located in the pancreatic tail (7/7); SPTs were located in the pancreatic tail in 14/21 (67%) and in the pancreatic body in 7/21 (33%).

For arterial-phase enhancement on contrast-enhanced CT (evaluable in 6/7 IPAS cases), IPAS showed heterogeneous enhancement in 6 cases; SPTs showed heterogeneous enhancement in 18/21 (86%) and homogeneous enhancement in 3/21 (14%). For CT attenuation relative to the spleen (evaluable in 4/7 IPAS cases), IPAS showed attenuation similar to the spleen in 2/4 cases, higher than the spleen in 1/4, and lower than the spleen in 1/4; all SPTs showed attenuation lower than that of the spleen (21/21). For calcification, no calcification was observed in the IPAS group; calcification was present in 12/21 (57%) of SPTs. For splenic-vessel adjacency, all IPAS lesions in this cohort were adjacent to splenic vessels (7/7); SPTs showed splenic-vessel contact in 10/21 (48%). Regarding margin definition, all IPAS lesions in this cohort had well-defined margins (7/7); SPTs had well-defined margins in 20/21 (95%). No pancreatic duct dilation was observed in either group in this cohort. For pathological maximum diameter, the median was 1.75 cm (IQR, 1.4–2.1) for IPAS and 3.0 cm (IQR, 2.6–4.5) for SPTs. Regarding consistency, IPAS lesions were soft in 4/6 cases; SPTs were soft in 4/21 (19%), intermediate in 7/21 (33%), and hard in 10/21 (48%). For gross color, all IPAS lesions in this cohort were reddish-brown (6/6); SPTs were reddish-brown in 6/21 (29%), grayish-white or yellow in 9/21 (43%), and mixed in 6/21 (29%). Regarding pathological margin definition, all IPAS lesions in this cohort had well-defined margins (6/6); SPTs had well-defined margins in 20/21 (95%). For imaging-measured lesion size, the median was 2.0 cm (IQR, 1.4–2.6) for IPAS and 3.2 cm (IQR, 2.5–3.9) for SPTs.

In summary, IPAS in this cohort most commonly presented as a pancreatic tail lesion with heterogeneous arterial-phase enhancement, CT attenuation approximating that of the spleen, adjacency to splenic vessels, and gross features of a solid, reddish-brown, soft mass with well-defined margins. These observed characteristics in our cohort may serve as reference points for the differential diagnosis of PNET and SPT. However, given the limited sample size of IPAS, these observations require validation in larger series. The summary of the efficacy of using different diagnostic or intervention methods to diagnose IPAS, PNET and SPT is presented in Table 6.

**Table 6:** Various diagnostic modalities and efficacies for PNET, SPT, and IPAS.

Examination modalities	PNET	SPT	IPAS
US	Ineffective	Ineffective	Ineffective
NC-CT	Equivocal	Equivocal	Ineffective
CE-CT/CE-MRI	Equivocal	Equivocal	Equivocal
CE-US	Equivocal	Equivocal	Effective
Tc-99m HDRBC scintigraphy	Ineffective	Ineffective	Effective
SPIO-enhanced MRI	Ineffective	Ineffective	Effective
^68^Ga-DOTA-SSA PET/CT	Effective	Ineffective	Ineffective
^111^In-Pentetreotide SPECT/CT	Effective	Ineffective	Ineffective
FNA	Effective	Effective	Effective
Surgery	Effective	Effective	Effective

Based on qualitative visual consensus, “Effective” indicates that the modality may facilitate diagnosis or characterization; “Equivocal” indicates limited or non-specific diagnostic value; and “Ineffective” indicates that the modality is generally insufficient for definitive differentiation.

68 Ga-DOTA-SSA PET/CT , Gallium-68 DOTA-Conjugated Somatostatin Analog Positron Emission Tomography/Computed Tomography; ^111^In-Pentetreotide SPECT/CT , Indium-111 Pentetreotide Single-Photon Emission Computed Tomography/Computed Tomography; CE-CT , Contrast-Enhanced Computed Tomography; CE-MRI, Contrast-Enhanced Magnetic Resonance Imaging; CE-US , Contrast-Enhanced Ultrasonography; FNA, Fine-Needle Aspiration; IPAS, Intrapancreatic Accessory Spleen; NC-CT , Non-Contrast Computed Tomography; PNET, Pancreatic Neuroendocrine Tumor; SPIO-enhanced MRI, Superparamagnetic Iron Oxide-Enhanced Magnetic Resonance Imaging; SPT, Solid Pseudopapillary Tumor of the Pancreas; Tc-99m HDRBC scintigraphy, Technetium-99m Heat-Denatured Red Blood Cell Scintigraphy; US , Ultrasonography.

## 4 Discussion

IPAS is frequently misdiagnosed as PNET, one of the most critical differential diagnoses (10, 11). The key points for differentiating IPAS from PNET have been summarized in Figure 4. PNET is an uncommon pancreatic lesion. PNET may present with functional symptoms depending on hormonal activity, whereas IPAS is usually asymptomatic. However, several patients with IPAS in our cohort presented with symptoms suggestive of PNET, illustrating the potential for clinical overlap. Despite being divided into functional and non-functional types, PNET overall has a higher symptom incidence than mostly asymptomatic IPAS (12, 13). However, over half of our IPAS patients had PNET-related symptoms—an overlap in clinical phenotype, which may be a key cause of their preoperative misdiagnosis as PNET. This may be a key reason for their preoperative diagnosis of PNET and subsequent surgical intervention. Combined with our findings and previous reports, IPAS mostly occurs in the pancreatic tail. Although PNETs can occur at any pancreatic site, contemporary data from large institutional series and the National Cancer Institute's Surveillance, Epidemiology, and End Results (SEER) program demonstrate a body and tail predominance (14). Accordingly, anatomic location alone is insufficient for reliable discrimination, necessitating the incorporation of complementary imaging and clinical features into the diagnostic assessment. Regarding imaging examinations, CE-CT represents an effective method for differential diagnosis. Owing to abundant vascularity, PNET manifests as enhancing nodules in both the arterial and venous phases (15). Notably, the splenic vessels represent a novel diagnostic insight: IPAS is located closer to the splenic vessels than PNET. This anatomical relationship may reflect the close developmental and vascular association between accessory splenic tissue and the splenic vasculature. However, unlike CE-CT, PNET and IPAS exhibit similar features on MRI, posing certain challenges for differential diagnosis (4). Diffusion-weighted imaging (DWI) represents the most robust validated noninvasive discriminator in this setting, with IPAS demonstrating splenic isointensity in 100% of cases compared with only 27% of small pancreatic tumors, yielding reported visual accuracies of 90%–95% (16). Contrast-enhanced endoscopic ultrasound (CE-EUS) has emerged as a valuable adjunct; IPAS typically manifests as a well-circumscribed, hypoechoic solid lesion with homogeneous enhancement, frequently demonstrating a vascular pedicle to the splenic hilum (17). Notably, PNETs are amenable to more specific functional imaging, including ^68^Ga-DOTA-somatostatin analogue PET/CT and ¹¹¹In-pentetreotide SPECT/CT (18). Furthermore, endoscopic ultrasound-guided fine-needle aspiration (EUS-FNA) can provide confirmatory diagnosis (19). However, clinicians should remain cognizant that both EUS-FNA without CD8 immunostaining and ^68^Ga-DOTATATE PET have been documented to yield false-positive PNET diagnoses in IPAS, as accessory splenic tissue may express somatostatin receptors and exhibit cytologic features that mimic PNET (20–22).

**Figure 4:**
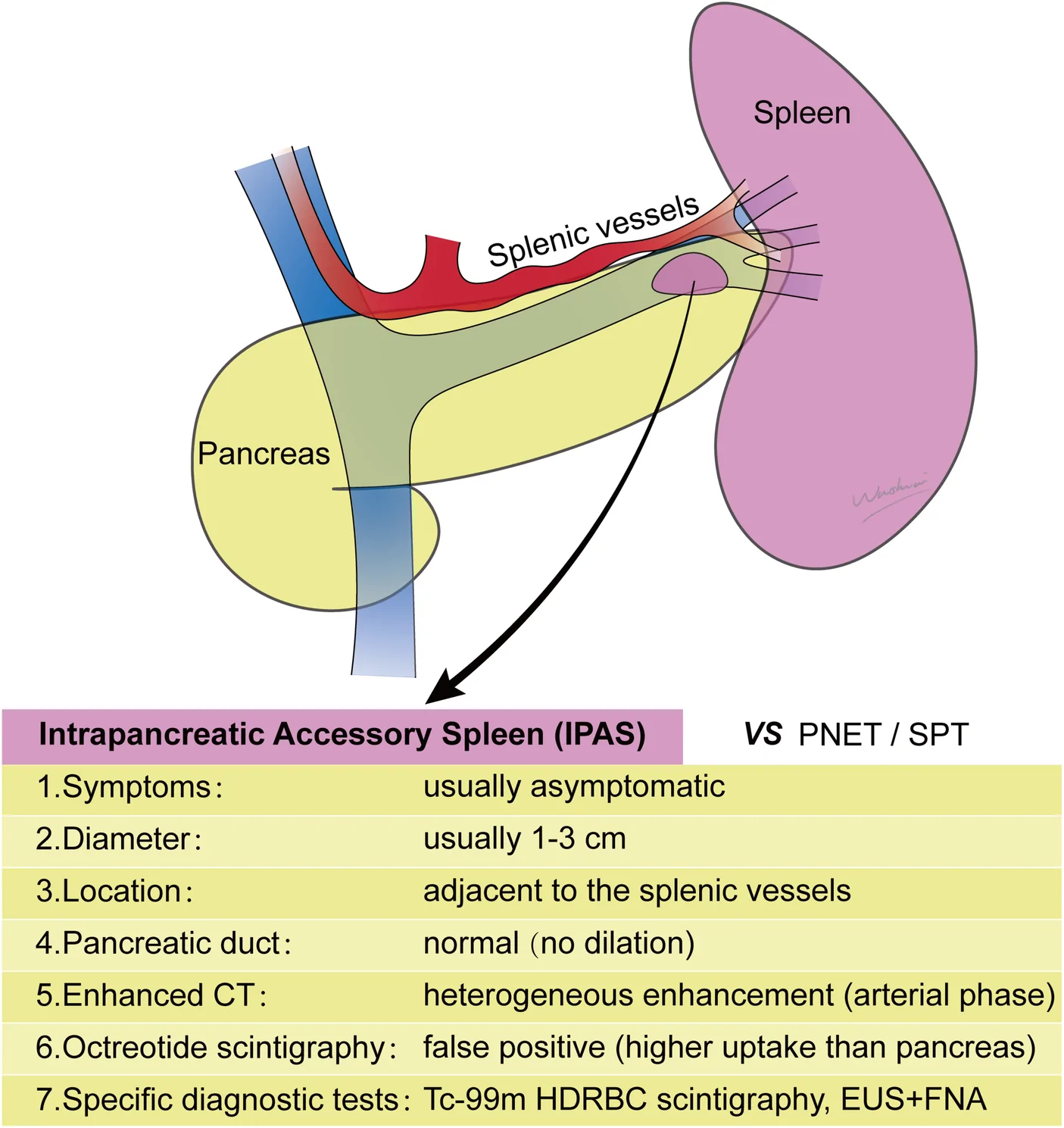
Schematic illustration of key distinguishing features among IPAS, PNET, and SPT.

SPT is another important differential diagnosis of IPAS. Figure 4 summarizes the key differential diagnostic points among IPAS, SPT, and PNET. SPT is predominantly seen in young women. Unlike PNET, SPT rarely presents with specific clinical syndromes. Symptomatic cases are mostly caused by mass effect (3, 23–25). This is also consistent with the findings of our study, where it is difficult to distinguish IPAS patients from SPT patients solely based on clinical symptoms. Clinically, the characteristic of SPT occurring predominantly in young women can definitely be regarded as an important point to distinguish it from IPAS in practice. Our data revealed a larger median lesion size for SPT compared with IPAS, a finding consistent with prior reports (26, 27). Whereas IPAS is predominantly encountered in the pancreatic tail, SPT may arise in either the body or tail, yielding distinct distributions. Moreover, although enhancement patterns on CE-CT may overlap, attenuation relative to the spleen and the presence of calcification differ markedly: IPAS more closely approximated splenic attenuation and lacked calcification, whereas all SPT lesions attenuated below splenic parenchyma and more than half harbored calcification (26). Cystic degeneration represents a hallmark of SPT. Pathologically, SPT is characteristically hemorrhagic and variegated on cut surface, displaying a complex admixture of solid, cystic, and hemorrhagic components rather than a homogeneous gray-white appearance (26, 28). Similarly, adjacency to the splenic vessels is a defining morphologic feature of IPAS, one that is less consistently observed with SPT. Unfortunately, SPT lacks highly specific noninvasive diagnostic modalities; endoscopic ultrasound-guided fine-needle aspiration (EUS-FNA) remains a feasible diagnostic approach (29, 30).

Reported incidence rates of IPAS vary across different studies. For example, data of 0.7% and 0.2% both indicate that IPAS is a rare lesion (31, 32). Owing to its low incidence, current research on IPAS has been largely confined to case reports and case series. This study summarized the clinical features, imaging and pathological data of all IPAS patients in our center. Our results indicated negligible gender differences in IPAS occurrence, which was inconsistent with Li et al.'s report of a female predominance, whereas the age distribution was generally consistent with their findings (33). This gender difference may be related to sample size or genetic background of regional populations. While literature has reported that accessory spleen (AS) with a diameter exceeding 4 cm in the absence of main splenic enlargement is rare, a case of IPAS measuring 5 cm in diameter was identified in our study, which may provide novel insights into IPAS (1). It has clinical implications. When there is a pancreatic tail mass larger than 4 cm, IPAS should also be considered in the differential diagnosis. Ordinarily, the majority of IPAS patients are asymptomatic, with the lesions incidentally detected (2). Notably, in our center, only two patients were asymptomatic; all others had PNET-related symptoms or abdominal discomfort (13). This discrepancy may be related to the fact that cases in this center were mostly from surgical cohorts. MRI findings showed that IPAS signal intensity was mostly comparable to or higher than normal pancreatic parenchyma on T1WI, inconsistent with Kim et al.'s report of IPAS hypointensity on T1WI (34). This may be related to differences in MRI field strength and scanning sequences. However, IPAS consistently had higher signals than the pancreas on T2WI, which was in line with their results. On CE-CT, IPAS and the spleen showed nearly equal signal intensity and heterogeneous enhancement, which was fully consistent with prior studies (7, 35). Consistent with previous literature, our findings indicated that postoperative pathological examination of IPAS revealed round masses with soft or firm texture in the pancreatic tail, which exhibited benign pathological features (e.g., clear circumscription and absence of perilesional invasion or compression) (35, 36).

Even among experienced clinicians, distinguishing IPAS from hypervascular pancreatic neoplasms remains challenging, as conventional CE-CT and MRI do not invariably permit reliable differentiation (35, 37). Accordingly, tissue-specific ancillary imaging modalities may offer substantial diagnostic value in cases where conventional imaging findings are atypical or diagnostic uncertainty persists. Technetium-99m heat-damaged red blood cell (Tc-99m HDRBC) scintigraphy represents a highly specific nuclear medicine technique for detecting splenic tissue, based on the selective sequestration of heat-damaged erythrocytes by the spleen; approximately 90% of injected HDRBCs are reportedly sequestered by splenic tissue, thereby enabling splenic-specific imaging (34). However, given the relatively limited spatial resolution of SPECT/planar imaging, small IPAS lesions may escape detection; hybrid imaging combining Tc-99m HDRBC SPECT with cross-sectional modalities such as CT or MRI may therefore enhance lesion localization and diagnostic confidence (34, 35).

Superparamagnetic iron oxide (SPIO)-enhanced MRI offers an alternative tissue-specific imaging approach with considerable diagnostic utility. SPIO particles are phagocytosed by reticuloendothelial macrophages, particularly within the spleen and liver, producing signal characteristics in IPAS that mirror those of native splenic tissue and thereby facilitating discrimination from surrounding pancreatic parenchyma and other pancreatic neoplasms (35, 38). Previous studies have demonstrated that SPIO-enhanced MRI enables definitive characterization of IPAS and serves as a valuable adjunct when nuclear medicine imaging is limited by suboptimal spatial resolution or equivocal findings (36, 39).

Beyond these modalities, contrast-enhanced endoscopic ultrasound (CE-EUS) constitutes an important ancillary diagnostic tool for IPAS. On conventional EUS, IPAS typically appears as a well-circumscribed, homogeneous hypoechoic lesion with echotexture resembling that of the spleen; on CE-EUS, it characteristically demonstrates marked, homogeneous hyperenhancement during the arterial phase, contrasting distinctly with the surrounding pancreatic parenchyma (40, 41). Recent investigations have further suggested that contrast-enhanced harmonic EUS (CH-EUS) may facilitate differentiation between IPAS and non-functional PNET: IPAS tends to sustain a vascular enhancement pattern paralleling that of the spleen, whereas PNET more frequently exhibits early washout (42).

Conversely, although ^68^Ga-DOTATATE/DOTATOC PET/CT represents an important functional imaging modality for evaluating somatostatin receptor-positive neuroendocrine tumors, its application in IPAS differentiation harbors potential pitfalls. Because IPAS may demonstrate somatostatin receptor-mediated radiotracer uptake, intense ^68^Ga-DOTATATE/DOTATOC avidity mimicking that of PNET may occur, leading to false-positive interpretations (21, 22). Therefore, in hypervascular lesions of the pancreatic tail, a positive ^68^Ga-DOTATATE/DOTATOC PET/CT should not be considered diagnostic of PNET in isolation; correlation with enhancement characteristics relative to the spleen on CT/MRI, together with confirmatory Tc-99m HDRBC splenic scintigraphy/SPECT when indicated, may improve the accuracy of distinguishing IPAS from PNET (43).

This study described the clinical characteristics of IPAS patients in our center. We compared the clinical features, imaging findings and pathological results of IPAS patients with those of PNET and SPT patients, respectively. However, this study has several limitations. First, the small sample size may compromise our statistical power and the robustness of the results. Our IPAS data were derived from a single center, and some patients lacked more detailed clinical data, such as tumor markers; collaboration with other centers may serve as an effective solution to these issues. Second, due to the limitations of retrospective research, as well as the low incidence and insidious clinical manifestations of IPAS, comprehensive imaging data were limited, highlighting the urgent need for prospective diagnostic studies.

## Data Availability

The original contributions presented in the study are included in the article/Supplementary Material, further inquiries can be directed to the corresponding author/s.
